# Multivariate regression modelling for gender prediction using volatile organic compounds from hand odor profiles via HS-SPME-GC-MS

**DOI:** 10.1371/journal.pone.0286452

**Published:** 2023-07-05

**Authors:** Chantrell J. G. Frazier, Vidia A. Gokool, Howard K. Holness, DeEtta K. Mills, Kenneth G. Furton

**Affiliations:** 1 Department of Chemistry and Biochemistry, Global Forensic and Justice Center, Florida International University, Miami, FL, United States of America; 2 Department of Chemistry and Food Science, Currently at Framingham State University, Framingham, Massachusetts, United States of America; 3 Currently at Lawrence Livermore National Laboratory, Physical and Life Sciences Directorate, Nuclear and Chemical Sciences Division, Livermore, CA, United States of America; 4 Department of Biological Sciences, Global Forensic and Justice Center, Global Forensic and Justice Center, Florida International University, Miami, FL, United States of America; University of Pisa, ITALY

## Abstract

The efficacy of using human volatile organic compounds (VOCs) as a form of forensic evidence has been well demonstrated with canines for crime scene response, suspect identification, and location checking. Although the use of human scent evidence in the field is well established, the laboratory evaluation of human VOC profiles has been limited. This study used Headspace-Solid Phase Microextraction-Gas Chromatography-Mass Spectrometry (HS-SPME-GC-MS) to analyze human hand odor samples collected from 60 individuals (30 Females and 30 Males). The human volatiles collected from the palm surfaces of each subject were interpreted for classification and prediction of gender. The volatile organic compound (VOC) signatures from subjects’ hand odor profiles were evaluated with supervised dimensional reduction techniques: Partial Least Squares-Discriminant Analysis (PLS-DA), Orthogonal-Projections to Latent Structures Discriminant Analysis (OPLS-DA), and Linear Discriminant Analysis (LDA). The PLS-DA 2D model demonstrated clustering amongst male and female subjects. The addition of a third component to the PLS-DA model revealed clustering and minimal separation of male and female subjects in the 3D PLS-DA model. The OPLS-DA model displayed discrimination and clustering amongst gender groups with leave one out cross validation (LOOCV) and 95% confidence regions surrounding clustered groups without overlap. The LDA had a 96.67% accuracy rate for female and male subjects. The culminating knowledge establishes a working model for the prediction of donor class characteristics using human scent hand odor profiles.

## Introduction

### Relevance of the study

Criminal activities involving robberies, assaults (sexual, simple, or aggravated), and rape are often executed with the use of the perpetrator’s hands. As a result, hands are a focal point of investigations as contributors of trace amounts of evidence that can be deposited on everyday objects through touch interactions. There is an exchange of both biological and inorganic material between the perpetrator and the crime scene during these interactions. In accordance with *Locard’s Exchange Principal*, the perpetrator will leave behind trace evidence in these moments [1–3]. Fingerprints and DNA are the biometrics most commonly utilized to identify a suspect or victim of a crime. However, these forms of evidence can be found in quantities that are too small to be used, leaving little to no forensic evidence that can be used for prosecution. Even in these instances where no physical fingerprint or DNA evidence is found, human scent evidence may still be recovered and used as an individualizing feature in an investigation. Though previous works have revealed this level of class characterization using an individual’s human scent from either breath or axilla, this study expands on this capability using hand odor which may be of great forensic value [4, 5].

Human odor is a complex mixture of volatile organic compounds (VOCs) secreted from the body that are impacted by host genetics, environmental factors, and physiological secretions [6]. VOCs are organic compounds, often with high vapor pressures, that are emitted into the environment as gases. The persistence of an individual’s odor in the environment is attributed to the constant shedding of the epidermis (outer layer) of the skin; this process leaves epithelial cells in the environment, along with sweat, oils, and other glandular secretions [7, 8]. Many compound classes are present in human emanations, including acids, alcohols, aldehydes, hydrocarbons, esters, and ketones [3].

An individual’s odor is comprised of primary, secondary, and tertiary odors [3, 8, 9]. Primary odor has been determined to be stable over time and distinct to an individual. One contributing factor to this distinctiveness is attributed to a polymorphic gene family known as the Major Histocompatibility Complex (MHC). The MHC contribution to human odor has been explained in three hypotheses. (1) The first hypothesis focuses on the presence of MHC-produced molecules found in sweat. (2) The second hypothesis states that MHC molecules may bind to specific peptides and present them to the surface of the cell/tissue and that these volatile metabolites may be the origin of skin odor VOCs. MHC molecules would therefore act as "odor carriers," with peptides functioning as precursors of VOCs [9]. (3) A final hypothesis suggests that MHC proteins/ peptides/metabolites may have a direct influence on the microbial biota. It is likely a combination of these hypotheses that truly explains how the MHC contributes to various roles in human odor production.

The genetic influence on microbial diversity leads to microbiota being another contributory factor of primary odor. As stated in a study by Shelley et. al, human perspiration has no odor until the microbiota in the skin begin to break down non-volatile chemicals into volatile molecules that are distinctive of human ’scent’ [10]. Although genetics and microbial diversity aid in the stability of odor, there is a secondary odor composition that has been determined to be variable and endogenous via the skin’s multi-layer composition. Physiological secretions produced from the dermis to epidermis are excreted through three types of glands: eccrine, apocrine, and sebaceous. Eccrine glands are distributed all over the human body but more densely in the palms, forehead, and soles of the feet, which is the focus of this study [11]. Tertiary odor has the highest variability due to exogenous compounds such as non-resident bacteria, cosmetic products, soaps, and perfumes.

Well-trained canines (*C*a*nis familiaris*) operate as specialized sentient detectors able to distinguish and identify personal human odor and other chemicals of interest [12–14]. Though canines have been proven to reliably identify persons based on their odor profile, laboratory based subject identification using analytical instruments has been difficult due to the lack of robust datasets and sufficiently developed analytical techniques. This work demonstrates the ability to predict donor gender based upon the VOCs present in a collected hand odor sample via Headspace-Solid Phase Microextraction-Gas Chromatography-Mass Spectrometry (HS-SPME-GC-MS). SPME is a solvent free method that integrates sampling, extraction, and concentration of analytes [15]. Existing human odor research has identified VOCs of interest that are characteristic of gender [16, 17], age [18], and racial/ethnic groupings [5, 19, 20]. These works indicated a capacity for predictive classification of individuals using hand odor samples.

This study demonstrates the efficacy of applying VOC profiles to the tasks of gender discrimination, comparison, and/or classification. Our analyses utilized an analysis of relative composition using targeted analyte and untargeted analysis of present VOCs paired with three linear regression modelling approaches.

These supervised approaches for the classification of donor gender, create a potential route to identifying donor characteristics based upon collected human hand odor samples. Within this exploration of supervised modelling approaches, the utility of an untargeted analysis of human odor VOCs was investigated. This technique monitored the response of collected VOCs from an untargeted approach to retrieve information that may normally be overlooked using a targeted approach alone. The specific presence and abundance of VOCs in human hand odor creates a chemical profile that can characteristically be applied to classify individuals based on class characteristics, increasing the utility of human hand odor when other biological evidence is lacking.

## Materials and methods

### Cohort

Sixty subjects of African American, Hispanic, and Caucasian race/ethnicity, between the age of 18–46 years old volunteered to participate in this study (Table 1). Before participation in the study, all subjects signed a consent form and filled out a questionnaire providing information about race/ethnicity, country of origin, gender, age, diet restrictions, and current state of health at time of sampling. The sampling procedures and protocols involving human subjects were authorized by Florida International University’s Institutional Review Board (IRB-19-0277) prior to commencement of the study. This study was conducted on a strict volunteer basis, therefore no participants received compensation.

**Table 1:** Demographics of subjects under study.

Race/ethnicity	Female	Male
African American	10	10
Hispanic	10	10
Caucasian	10	10

### Chemical and materials

Methanol (Fisher Scientific, Pittsburgh, PA) was used for the pretreatment of the collection material, 2 in. x 2 inch., 12ply, 100% cotton, sterile gauze pads (DUKAL Corporation, Syosset, NY). The collection vessel for hand scent samples were 10-mL, clear, screw top glass vials with PTFE/Silicone septa, respectively (SUPLECO, Bellefonte, PA). 10-mL vials were placed in Fisherbrand^TM^ Isotemp^TM^ Digital Dry Bath Incubators for HS-SPME equilibration and extraction. The Solid Phase Microextraction (SPME) fibers used were 24ga 50/30μm Divinylbenzene/Carboxen/Polydimethylsiloxane (Grey) (SUPELCO, Bellefonte, PA).

### Collection material pretreatment

Although human collection materials were biologically sterile, the absorbent material contained compounds such as Decanal and Nonanal, also found in human hand odor [21]. The storage vials and cotton gauze underwent a pretreatment process to remove any background interferents. The 10-mL vials and caps were cleaned with a mildly basic soap solution (Contrex AL®, Decon Labs, Inc.), rinsed with warm tap water, followed by deionized (DI) water, and a final acetone rinse, before baking in an oven at 105°C for 1 hour. The cotton gauze was pre-treated by laying the gauze flat on a sterilized watch glass, spiking the gauze pad with 1mL of methanol, placing the gauze in a cleaned 10-mL vial, and baking in an oven at 105°C for one hour [22]. The background levels of VOCs on the pretreated cotton gauze and vial were monitored by HS-SPME-GC-MS prior to use in sample collection.

### Hand odor sample collection and analysis

The hand odor collection protocol was modified from the original protocol published by Curran et al. in efforts to resemble a more realistic collection of hand volatiles [3]. Each subject was instructed to *not* wash their hands for a minimum of one hour prior to sampling. Hand odor was collected from each subject while sitting indoors. A pretreated 2 in. x 2 in., 12ply, 100% cotton DUKAL gauze was swiped on each palm of the hands and then squeezed between the palms for 10 min. Upon completion of the 10 min hold, the sampled gauze was placed back into its respective 10 mL vial and capped. The 10 mL vial containing sampled gauze was placed into a digital bath (set to 50°C) for 24 hours. After 24 hours, a clean 50/30μm DVB/CAR/PDMS (Grey) SPME fiber was placed into the sample vial and exposed for 15 hours. After exposure, the SPME fiber was subsequently desorbed into the inlet of an Agilent 6890 GC coupled with an Agilent 5973 MSD. Fibers were desorbed at 250°C for (5) minutes in the GC inlet. A SolGel-WAX^TM^ 30M x 0.25mm ID x 0.25μm phase thickness (SGE Analytical Science) column was used with ultra-high purity helium (Airgas) as the carrier gas (S1 Table). The method was optimized with a 36-standard mixture of known human scent compounds and continuously ran with each set of samples to monitor the performance of the GC-MS.

### Data pre-processing

The collected GC-MS data files for the 30 male and 30 female subjects were aligned by retention time and evaluated for reoccurring peaks present in the total ion chromatogram (TIC). All compounds were assigned numbers instead of names and corresponding peak areas were organized into tables for supervised dimensional reduction analysis. This pre-processing feature can be completed manually or with the aid of commercially available software products such as Sepsolve’s ChromCompare program. The current dataset was retention time aligned using a bin size of 0.040 minutes, the procedure was conducted using a custom script in R, a similar approach has been used in the pre-processing of other HS-SPME-GC-MS collected human odor datasets [23, 24].

### Partial Least Squares- Discriminant Analysis (PLS-DA)

Partial Least Squares- Discriminant Analysis (PLS-DA) is a latent variable regression method based on covariance between the predictors and the response [25]. PLS-DA represents a quantitative relationship between a matrix, X, usually comprising spectral or chromatographic data of a set of calibration samples, and another matrix, Y, containing quantitative values [26]. This supervised method uses multivariate regression techniques to extract, via a linear combination of the original variables, information that can predict class membership [27]. The pre-processed data containing aligned peak areas were log_10_ transformed and auto-scaled (mean-centered and divided by the standard deviation of each variable); these formatted data were analyzed using MetaboAnalyst software version 5.0 (https://www.metaboanalyst.ca). The implementation of this method, as described by Wehrens et al. produced a PLS-DA 2D and 3D model [28].

### Orthogonal-Projections to Latent Structures Discriminant Analysis (OPLS-DA)

Orthogonal-Projections to Latent Structures Discriminant Analysis (OPLS-DA) is a supervised dimensional reduction tool. The orthogonal methodology is superior for predicting variables contributing to class separation when compared to techniques such as PLS-DA [29]. OPLS separates the systematic variation in X into two parts, one that is linearly related to Y and one that is unrelated (orthogonal) to Y [26]. The algorithm is modified to model separately the variations of the predictors correlated and orthogonal to the response, minimizing two or more predictive components to a single predictive component [30]. The log_10_ transformed data set was further analyzed in MetaboAnalyst software version 5.0 (https://www.metaboanalyst.ca) using the OPLS-DA algorithm as described by Thévenot et al. [25]. Leave one out cross validation (LOOCV) ran concurrently with the OPLS-DA algorithm to ensure data were not overfitted within the model. The model produced a resulting graph that demonstrated a significant difference among the predictive component and its orthogonal response to separate male and female subjects.

### Linear Discriminant Analysis (LDA)

Linear Discriminant Analysis (LDA) is a supervised learning technique that searches for those vectors in the underlying space that best discriminate among classes (rather than those that best describe the data) [31]. In its application to the present dataset, LDA was used for dimensional reduction and supervised modelling of gender classification (Female or Male). Prior to the pre-processed data submission to JMP®, Version 16.1.0. SAS Institute Inc., Cary, NC, 1989–2021, the processed files were filtered to contain all peaks present in more than 20% of submitted samples (12/60), as determined through retention time-based peak matching. The peak areas of the aligned samples were log_10_ transformed; the LDA model was validated using LOOCV. The resulting graph demonstrated separation of male and female subjects and the predictive ability of the model via cross-validation.

## Results and discussion

**Fig 1:**
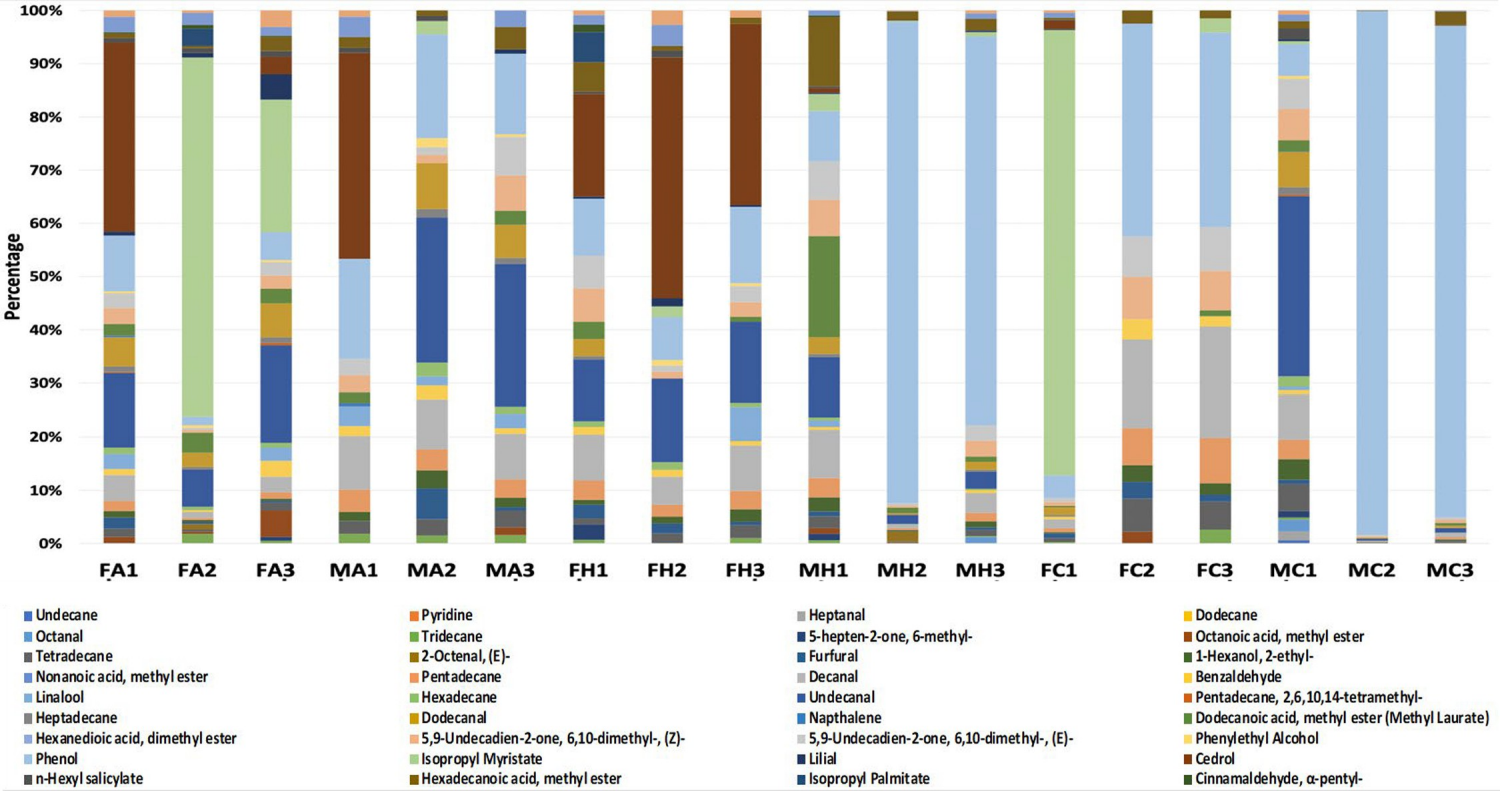
Subset of VOC profiles. Targeted approach visualization of nine females and nine males from each ethnicity (F = Female, M = Male, A = African American, C = Caucasian, H = Hispanic) VOC odor profiles.

The exploration of human odor to discriminate individuals through various class characteristics (gender, race/ethnicity, etc.) for forensic application has been conducted analytically and biologically via GC-MS and canines, respectively. Various studies have examined and detected (in the headspace profiles) the presence of several compounds that have been articulated as human scent compounds [21]. However, the historical analysis of hand odor profiles has been constricted to a targeted approach with visualization of abundances with known human scent standards (stacked bar graphs), unsupervised multivariate analysis (i.e., PCA), and a minute exploration of linear discriminant analysis. Discrimination amongst gender was observed in these previous studies, although this was contingent upon the standard mixture ran for those specific analyses or body sites with these compounds of interest varying between publications. In this work, a targeted approach was conducted on a subset (18/60) of nine male and nine female samples to display both the individualizing capability of VOC profiles and its limiting factor, without prior information of the collected sample, there is no class characteristic classification/identification made apparent by such visualization (Fig 1).

### Partial Least Squares- Discriminant Analysis (PLS-DA)

**Fig 2:**
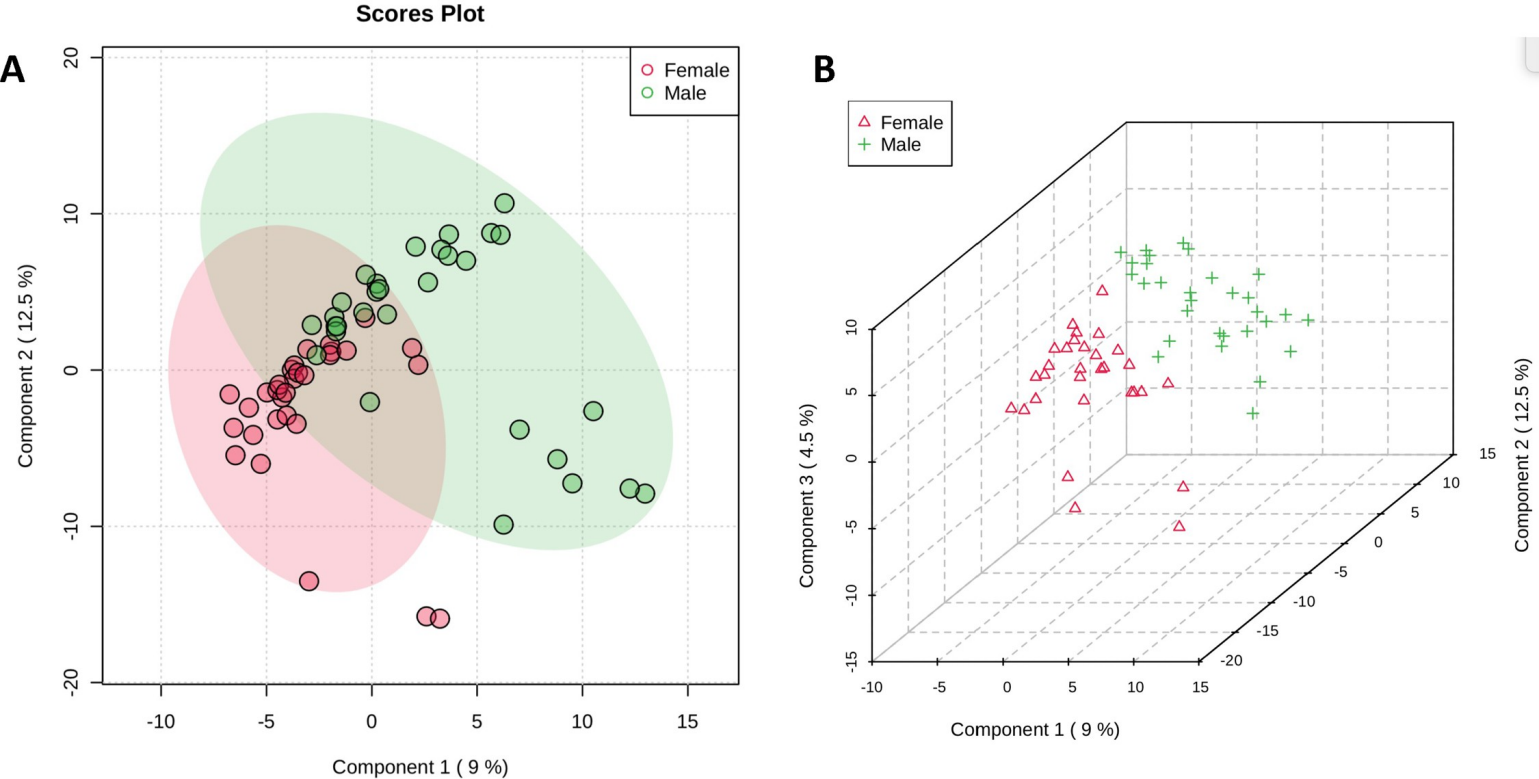
PLS-DA scores plots. (A) 2D PLS-DA scores plot showing clustering but no class separation of HS-SPME-GCMS VOCs from Female and Male subjects. (B) 3D PLS-DA scores plot displays clustering and minimal class separation of HS-SPME-GCMS VOCs from female and male subjects.

Various studies have examined and detected (in the headspace profiles) the presence of several compounds that have been articulated as human scent compounds [21]. The 2D scores plot (Fig 2A) was comprised of the two principal components that illustrated clustering of the male and female subjects but no separation of the two classes. The green and red ellipses surrounding the male and female clusters represent the 95% confidence region. The same principal was applied to the 3D scores plot, the incorporation of a third component revealed clustering of the male and female subjects with separation of the two classes (Fig 2B). The produced 2D PLS-DA (two component) and 3D PLS-DA (three component) score plots were created using log-transformed TIC peak areas.

While the PLS-DA model illustrates variations that may exist in the measurements for group prediction, these variations may be uncorrelated. Therefore, OPLS-DA is often implemented in order to disentangle group-predictive and group-unrelated variation in measured data [28]. Variable importance projections (VIP) scores plots for this model have been included as S1 Fig, this information indicates of the relative contributions of important compounds influencing a gender group classification decisions using the presented PLS-DA model.

### Orthogonal-Projections Latent Structures Discriminant Analysis (OPLS-DA)

**Fig 3:**
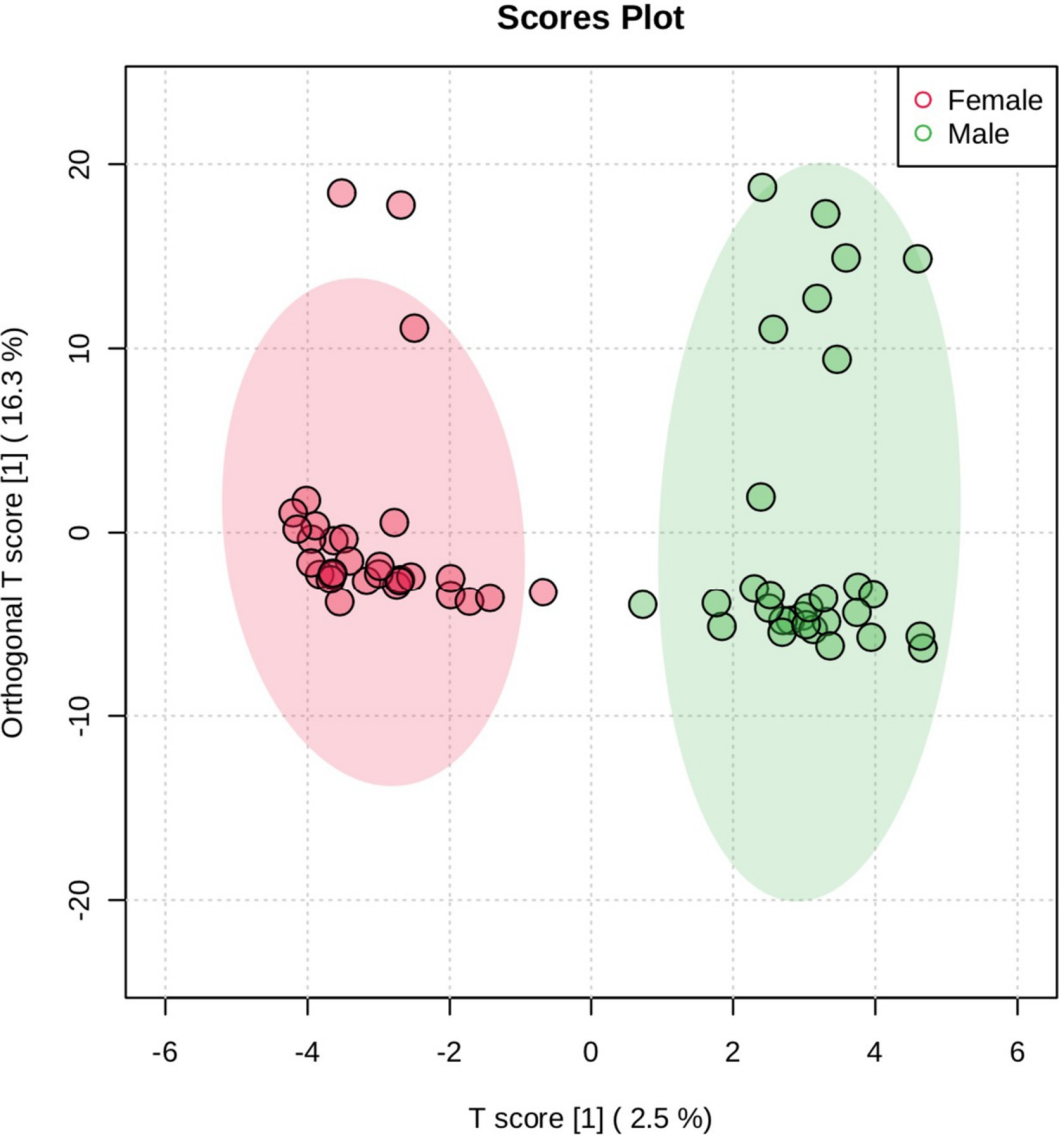
OPLS-DA scores plot. Principal components with highest variance show complete separation of Female and Male subjects.

The transformed peak data table was further analyzed to determine whether HS-SPME-GCMS could reveal class separation of odor profiles into male and female clusters. Components are contributed by VIPs, comprised of both the loading weights and the variability of the response explained [26]. The T-score [1] reflects the predictive component of the data set and orthogonal T-score [1] represents the component unrelated to predictive component [25]. The calculated covariance is explained within the respective parentheses (16.3% and 2.5%). The ellipses correspond to 95% of the multivariate normal distributions with the covariances for each class being shown. The supervised classification method OPLS-DA was employed utilizing all peak areas detected and the resulting graph demonstrated sufficient variations in the data, such that, clustering and separation of female and male subject data were observed without the requirement of compound identification (Fig 3). The potential applicability of rapidly running an odor sample and using these multivariate analyses could aid investigations to eliminate 50% of the population from subsequent considerations.

### Linear Discriminant Analysis (LDA)

**Fig 4:**
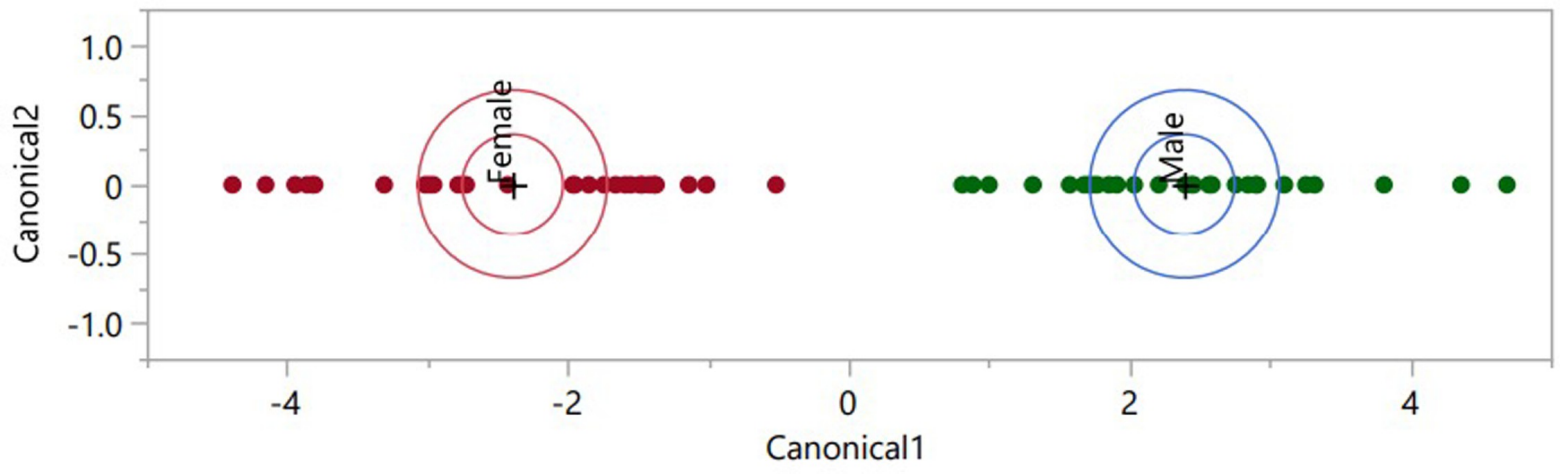
LDA. Class separation of HS-SPME-GC-MS VOCs from Female and Male subjects.

Linear discriminant analysis was used as a dimensional reduction and modelling technique for the predictive classification of donor samples into “Male” or “Female” classes. Due to the composition of the DVB/CAR/PDMS SPME fibers utilized and the makeup of the polar SolGel-WAX^TM^ capillary column, highly abundant siloxane peaks were observed in the obtained chromatograms and spectra. Peaks appearing in less than 20% and more than 95% of samples were removed to minimize the effect of background interferents. This procedure was chosen to remove both consistently present background signals and sporadically present interferents. The filtered peak table was log-transformed and used to create an LDA model, the model was validated using LOOCV. The classification and separation of male and female subjects illustrated by the LDA model (Fig 4) were cross-validated using leave-one-out cross-validation.

The LDA model’s performance was determined to have a 29/30 (96.67%) accuracy rate for predicting samples from male donors as male. The LDA model also predicted the source of female donated samples as female at a 29/30 (96.67%) accuracy rate. One male sample and one female sourced sample were misclassified using the produced model. The misclassification of the two samples could be correlated to the abundance of a specific or group of compounds resembling an abundance of a male subject profile and vice versa for a female subject. Further analysis of what compounds could be driving this has yet to be evaluated.

The overall impact of this study could assist in changing the trajectory of how we currently utilize human odor in the field of forensic. Investigators now have an opportunity to rapidly assess the volatiles of collected human odor samples. The limitations within this study correlate to the length of the HS-SPME-GC-MS total analysis time the researchers used an exhaustive method in terms of extraction and GC-MS run time to minimize the chances of overlooking informative larger compounds that could take longer to volatize or elute from the column to the detector. However, this method could be refined based on the findings of this study to allow for reduced collection analysis times. In addition, the sample size for this study was too small for machine learning techniques such as random forest and support vector machine models to be leveraged.

Both the untargeted and targeted approaches presented in this study have pros and cons. The standard targeted approach requires a standard mixture to match known human volatiles with volatiles in the sample. This inherently limits the number of compounds being monitored to those that have been previously identified and which can be attained as a reference quality standard. There is the chance of ignoring relevant information in a human odor sample because the compound is not present in the targeted list. Additionally, conducting a targeted search is a time- consuming process that includes constructing complex reference mixtures and multiple calibration curves. On the other hand, the real strength of the approach is the ability to quantify the abundance of volatiles and develop easily understood graphics that a jury can interpret (including odor expression “barcodes”). Looking to the untargeted approaches, the largest con is the inability to pointedly say what compound is driving the observed separation of classes. If the identity of a compound is known it begs to ask why the compounds aren’t initially monitored, transforming the approach into a targeted one. Untargeted and targeted approaches have complementary roles in this field of study with the boundaries of exploration into relevant compounds and abilities to differentiate samples types and classes being pushed by untargeted analyses and supported by the confirmation of a subsequently developed targeted approach.

## Conclusion

In this study, hand VOC odor profiles from 60 self-identifying male/female participants were evaluated with supervised multivariate regression models for gender classification. The partial least squares-discriminant analysis (PLS-DA) 2D model displayed lesser discrimination of gender but exhibited the ability to cluster female and male subjects. With the addition of a third component greater classification and discrimination of gender could be observed in the 3D model in comparison to the 2D model. The highest discrimination and classification of subject gender were observed with orthogonal projections latent structures- discriminant analysis (OPLS-DA) and linear discriminant analysis (LDA) as confidence level ellipses of both models were not seen to intersect. In comparison, stacked bar graphs displayed the individualization of VOC profiles but lack the capability to provide any additional pertinent information such as gender, if donor characteristics are not previously known. The cross validation of the LDA model demonstrated its efficacy with a 96.67% accuracy rate for both male and female samples. The partial least squares-discriminant analysis (PLS-DA) 2D model displayed lesser discrimination of gender but exhibited the ability to cluster female and male subjects. With the addition of a third component greater classification and discrimination of gender could be observed in the 3D model. This work assessed additional components of statistical analyses necessary for human scent research to be applied in support of forensic identification. Further work is needed in the exploration of the feature selection provided by the VIP scores and loading plots of the PLS-DA and OPLS-DA model that are indicative of compounds that aid in discrimination and gender prediction of hand odor profiles. The overall statistical workflow could be applied to other identification factors such as ethnicity/race and age when other discriminatory evidence (e.g., DNA) may be lacking. Overall, the application of the discussed models throughout this paper can be applied to various forensic data sets regardless of variation in parameters used for GC-MS data collection and paves way for a tool that can perform standardized VOC comparisons.

## Supporting information

S1 Table HS-SPME-GC-MS analytical parameters. The analytical parameters for the applied GC-MS method on an Agilent 6890 GC coupled with an Agilent5973 MSD for analyzing HS-SPME samples.
(PDF)

S1 Fig VIP scores plot. The PLS-DA VIP scores plot associated with the 60 subject sample set. Compounds are labeled using their associated retention time. The compounds’ influence determining each group classification are ranked low to high.
(PDF)
