# Supplementary material for: Multivariate regression modelling for gender prediction using volatile organic compounds from hand odor profiles via HS-SPME-GC-MS
Source: PLoS One. 2023 Jul 5;18(7):e0286452. doi: 10.1371/journal.pone.0286452 (PMC10321641; doi:10.1371/journal.pone.0286452)
Supplement: S1 Table — The analytical parameters for the applied GC-MS method on an Agilent 6890 GC coupled with an Agilent5973 MSD for analyzing HS-SPME samples. (PDF) [file pone.0286452.s001.pdf]

S1 Table . HS-SPME-GC-MS analytical parameters.

**HS-SPME**	
Fiber	Divinylbenzene/Carboxen/Polydimethylsiloxane (50/30µm)
Exposure time	15 hours
Exposure temperature	50°C
**Gas Chromatography**	
Column	SGE Analytical Science SOL-GEL-Wax™ (30m x 0.25mm, 0.25 µm)
Carrier gas	Helium (He)
Constant flow	1.0 mL/min
Inlet temperature	250°C
Ramp	40°C for 1.25 min<br>10°C/min until 135°C for 4 min<br>10°C/min until 185°C for 4 min<br>10°C/min until 205°C for 4 min<br>30°C/min until 280°C
Oven run-time	32 min
**Mass detector**	
Source temperature	230°C
Ionization mode	Electron Ionization
Scan range	40-400m/z

The analytical parameters of an Agilent 6890 GC coupled with an Agilent5973 MSD for HS-SPME samples.
