# Supplementary figures and images for: Multivariate regression modelling for gender prediction using volatile organic compounds from hand odor profiles via HS-SPME-GC-MS
Source: PLoS One. 2023 Jul 5;18(7):e0286452. doi: 10.1371/journal.pone.0286452 (PMC10321641; doi:10.1371/journal.pone.0286452)

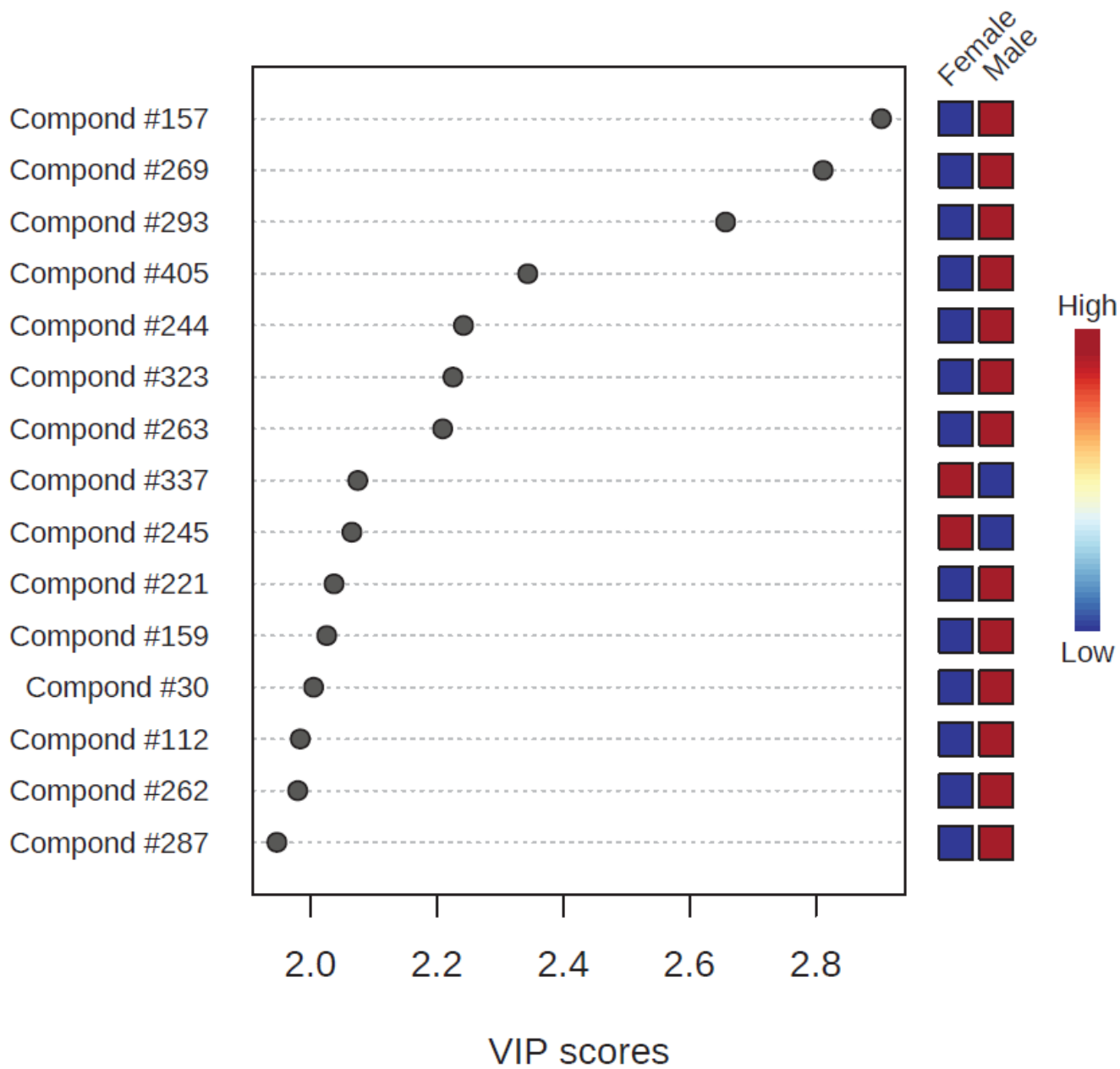

Supplement: S1 Fig — The PLS-DA VIP scores plot associated with the 60 subject sample set. Compounds are labeled using their associated retention time. The compounds’ influence determining each group classification are ranked low to high. (PDF) [file pone.0286452.s002.pdf]
